# The Association of Body Image Self-Discrepancy With Female Gender, Calorie-Restricted Diet, and Psychological Symptoms Among Healthy Junior High School Students in Japan

**DOI:** 10.3389/fpsyg.2021.576089

**Published:** 2021-10-05

**Authors:** Kyoko Nomura, Yuki Itakura, Sachiko Minamizono, Kazuyo Okayama, Yumiko Suzuki, Yukari Takemi, Akemi Nakanishi, Kumi Eto, Hitoshi Takahashi, Yuki Kawata, Hitomi Asakura, Yorika Matsuda, Naoko Kaibara, Sakiko Hamanaka, Hiroko Kodama

**Affiliations:** ^1^Department of Environmental Health Science and Public Health, Akita University Graduate School of Medicine, Akita, Japan; ^2^Faculty of Human Development and Culture, Fukushima University, Fukushima, Japan; ^3^Major in Health and Dietetics, School of Health Science, Teikyo Heisei University, Tokyo, Japan; ^4^Hiroshima University Graduate School of Humanities and Social Sciences, Hiroshima, Japan; ^5^Department of Nutrition Sciences, Kagawa Nutrition University, Saitama, Japan; ^6^Department of Health and Physical Education, Kokugakuin University, Kanagawa, Japan; ^7^Department of Nutrition, Teikyo University Hospital, Tokyo, Japan; ^8^Department of Health and Nutrition University of Human Arts and Sciences, Saitama, Japan

**Keywords:** body image self-discrepancy, eating disorder, adolescents, depressive mood, calorie-restricted diet

## Abstract

**Background:** Body image self-discrepancy reflects a preference for weight loss regardless of normal body size and is a distorted cognition that may be a precursor to eating disorders. The aim of this study was to investigate factors associated with body image self-discrepancy among healthy junior high school students in Japan.

**Method:** This cross-sectional study was conducted at one junior high school in Saitama, Japan, in December 2016. After excluding obese participants (defined as 20% above their ideal weight), 304 students (mean age, 13.9years; *n*=181 girls, 59.5%) who fell into underweight (*n*=22, 7.2%) and normal weight categories were selected. Body image self-discrepancy was measured using the Contour Drawing Rating Scale which includes eight separate figures representing body sizes. We then calculated the difference by subtracting ideal from current body sizes and defined body image self-discrepancy if the difference >1.

**Results:** Girls constituted 92% (n=49) of the 53 students with body image self-discrepancy. In all students, multivariable stepwise models demonstrated that female gender (OR, 6.92, 95% CI: 2.33–20.51), a calorie-restricted diet (OR, 5.18, 95% CI: 2.22–12.05), and psychological symptoms (OR, 1.47, 95% CI: 1.15–1.87) were significantly associated with an increased risk of body image self-discrepancy. Specifically for girls, an increased risk of body image self-discrepancy was associated with calorie-restricted suppers and psychological symptoms.

**Conclusion:** Body image self-discrepancy among healthy adolescents in Japan was found to be closely linked to being a girl, having a calorie-restricted diet, and having psychological symptoms.

## Introduction

Eating disorders were once regarded as a phenomenon specific to Western society; however, they now comprise a global issue, and a dramatic increase has been recorded across Asia’s high-income population of young women and girls. This increase has noted in Singapore, Hong Kong, Korea, and Taiwan (Chisuwa and O’Dea, 2010). Japan has also reported significant increases beginning in the 1990s (Tomifusa et al., 1996; Chisuwa and O’Dea, 2010), and according to a recent survey of seven prefectures of Japan, the prevalence of anorexia nervosa, a severe type of eating disorder, is estimated to range from 0.05 to 0.43% among girls in junior high school (Hotta et al., 2015).

Anorexia is known to be a comorbidity associated with mental disorders, including mood, depressive, and anxiety disorders (Yao et al., 2016), some personality disorders, and substance abuse disorders (Marucci et al., 2018). Hence, it is important to identify high-risk individuals early in life before subsequent symptoms and psychological comorbidities emerge (Arcelus et al., 2011). Individuals with eating disorders are also more likely to have cognitive distortions regarding food, weight, and body shape. For example, a core symptom of anorexia is body image self-discrepancy, which is an obsession with being overweight despite actually being substantially underweight.

According to a 2002 Japanese nutrition survey Ministry, Health, Labour, and Welfare, 2002), cognitive distortions regarding body shape and size in Japan are primarily observed among young women. The survey reported that 64.1% of girls aged 15–19years were trying to lose weight regardless of their current weight, and 40% of girls who were underweight were still trying to lose weight. A previous study (Suka et al., 2006) in Japan reported that body image self-discrepancy was also observed in a healthy young population of 5,244 preadolescents (2,452 boys and 2,792 girls aged 12–13years) and may have unfavorably modified their healthy behavior. The authors demonstrated that students who perceived themselves as overweight, regardless of their actual body size, had tried dieting more frequently than other students. A recent review found that though there is some degree of body image distortion among healthy individuals without eating disorders, individuals with eating disorders are likely to have particularly inaccurate perceptions of their bodies (Lantz et al., 2018). This finding suggests that healthy individuals with body image self-discrepancy are at risk of developing eating disorders through repetitive dieting. Given that the prevalence of eating disorders has increased quickly over the past three decades in Japan, it would be useful to identify the characteristics of healthy individuals with body image self-discrepancies. For example, we believe that the findings of this study may help identify individuals at high risk and introduce early interventions before clinical manifestations occur. In addition, the findings may expand the current understanding of the underlying mechanisms and pathology of eating disorders. Thus, the purpose of this study is to investigate factors associated with body image self-discrepancy among healthy junior high school students in Japan.

## Materials and Methods

### Participants

This cross-sectional study was conducted in December 2016 at one junior high school in Kumagaya City, Saitama prefecture, Japan, where students are 13 to 15years old. The study was conducted with a mutual understanding between the researchers and schoolteachers of the importance of nutrition and health education for young adolescents. Among the 552 recruited students, 535 (*n*=276 girls, 51.6%) answered a self-report questionnaire about body image and eating behaviors (response rate: 96.9%).

The exclusion criteria were as follows: students who (a) did not provide either their height (*n*=38) or weight (*n*=46) (b) fell into a range of 20% or more above their ideal bodyweight calculated by (current weight – ideal weight)/ideal weight (*n*=28) (c) had provided an unrealistic value for their height or weight (*n*=3), or (d) did not answer questions regarding current body image (*n*=77), ideal body image (*n*=92), or physical and psychological symptoms (*n*=1). Based on these criteria, 304 students (*n*=181 girls, 59.5%) were included for a final analysis.

This study was conducted according to the Declaration of Helsinki and approved by the ethics committee of Teikyo Heisei University. Written informed consent was obtained from all participating students and their parent(s)/guardian(s). The school provided the completed self-report questionnaire responses to the researchers as anonymized data.

### Measures

The questionnaire contained items related to the students’ basic characteristics (i.e., age and gender), weight, height, types of extracurricular activities (i.e., physical or non-physical activities), frequency of skipping breakfast, use of calorie-restricted diets, frequent physical and psychological symptoms, and self-esteem.

#### Body Image Self-Discrepancy

We used the Contour Drawing Rating Scale (Thompson and Gray, 1995), which consists of eight line-drawings of men’s bodies and eight of women’s bodies, separately arranged by sex and numbered from “1” to “8” (from the smallest body type to the largest). Participants chose the drawing they thought best matched their current body size and the one that matched their ideal body size. Using these responses, we calculated the difference by subtracting each participant’s chosen ideal from current body sizes; participants’ for whom the difference in the body sizes was greater than “1″ were defined as having a *body image self-discrepancy*, while “1″ indicates a body image self-accordance. Scores on this rating scale have been shown to have acceptable test–retest reliability and convergent validity (Gardner and Brown, 2010).

#### Unhealthy Eating Behaviors

The questionnaire included two items to examine unhealthy eating behaviors: (1) the number of times breakfast was skipped in a week and (2) the use of a calorie-restricted diet in the past month. Participants were first asked how many times per week they skipped breakfast; “skipping breakfast” was defined as not eating breakfast at all or eating breakfast six times or less (i.e., not every day) in a week. They were also asked whether they had restricted the amount of food they ate at any time in the past month to lose weight (*yes* or *no*). If they responded “yes,” they were asked which meal they had restricted (*breakfast*, *lunch*, *supper*, *sweets*, or *snacks*). Participants were considered to have a calorie-restricted diet if they had both restricted their food intake and, conditionally, restricted *supper*, as this meal is considered the one comprising the highest caloric intake.

#### Frequent Physical and Psychological Symptoms

Unlike most physical symptoms (headaches, stomachaches, sleepiness), the Diagnostic and Statistical Manual of Mental Disorders, Fifth edition (American Psychiatric Association, 2013) considers depression and fatigue to be individual components of the diagnostic criteria for a *mood disorder*; thus, we used the categories *physical symptoms*, including headaches, stomachaches, and sleepiness, and *psychological symptoms*, including fatigue and depressive mood. These five symptoms were assessed based on frequency in the past month using the following questions: “How often do you experience headaches?;” “How often do you experience stomachaches;” “How often do you experience fatigue (feel tired, exhausted);” “How often do you feel sleepy;” and “How often are you depressed or not eager to do anything?” Responses were provided using a four-point Likert scale: (4) for *very frequently*, (3) for *occasionally*, (2) for *rarely*, and (1) for *very rarely*. The scores were added separately for physical and psychological symptoms; the total score was used as a continuous variable, with higher scores indicating more frequent symptoms.

#### Self-Esteem

Referring to previous literature (Sabatella and von Wyl, 2019), we measured self-esteem using the following five validated and reliable items: “On the whole, I am satisfied with myself,” “I feel that I have a number of good qualities,” “I am able to do things as well as most other people,” “I feel that I’m a person of worth, at least on an equal plane with others,” and “I take a positive attitude toward myself.” We did not use all 10 items of the original scale, as the questionnaire had limited space; moreover, young adolescents would likely not understand some of the items. Hence, we selected five items that adolescents aged 13–15years could comprehend. Responses were provided using a four-point Likert scale: 4 for *strongly agree*, 3 for *agree*, 2 for *disagree*, and 1 for *strongly disagree*. For each respondent, the total score for self-esteem was calculated (possible range: 5–20) and used as a continuous variable, with higher scores indicating higher levels of self-esteem.

### Data Analysis

Participant characteristics were analyzed to identify differences between female and male students and between the presence or absence of body image self-discrepancy, using t-tests for continuous variables and chi-squared tests for categorical variables. Cronbach’s alpha, a measure of internal consistency, was calculated for self-esteem. Factors associated with body image self-discrepancy were investigated using logistic regression analysis. Odds ratios (OR) of body image self-discrepancy, defined as a difference between current and ideal body image >1, were calculated along with 95% confidence intervals (95% CI). Multivariable stepwise logistic regression modeling was used to identify predictor variables in the final models. The stepwise modeling process starts with a model with no variable and ends with the full model (all the predictor variables selected). We considered age, gender, extracurricular activities, skipping breakfast, a calorie-restricted diet, self-esteem, and physical and psychological symptoms as predictor variables. Accordingly, we listed all these variables in the statistical procedure “PROC LOGISTIC” statement embedded in the SAS software.

Sensitivity analysis was conducted to identify to what extent the significant variables associated with a difference between current and ideal body image >1 would change with a difference >2. Sensitivity analysis examines the degree to which the outcome will be affected when variables/parameters change.

The analyses were performed with all participants and stratified by gender. All analyses were conducted using SAS (9.4 version, CA, United States), with the significance level set at 5% (two-sided).

## Results

Table 1 shows participant characteristics. The mean age of the participants was 13.9±0.8years, and the difference in Contour Drawing Rating Scale scores, calculated by subtracting ideal body size from current body size, was higher among girls than boys (0.9 vs. 0.6, *p*<0.001). Girls constituted 92% of the students who had body image self-discrepancy (*n*=49). Boys were more likely than girls to participate in sports-related extracurricular activities after school (73% vs. 53%), whereas girls were more likely to participate in non-physical extracurricular activities (31% vs. 7%). Additionally, 16% of girls and 20% of boys reported participating in no extracurricular activities (*p*<0.001). Over one-third of girls had restricted their calorie intake to lose weight in the past month, which was considerably higher than that for boys (38% vs. 6%, respectively, *p*<0.001). Although not statistically significant, sleepiness and depressive mood were slightly higher among girls than boys (*p*=0.07), whereas one aspect of self-esteem, measured by the statement “On the whole, I am satisfied with myself,” was slightly higher among boys than girls (*p*=0.08).

**Table 1:** Subjects characteristics (*n*=304).

	All *(n*=304)		Female (*n*=181, 60%)		Male (*n*=123, 40%)		*p*
Grade, n (%)							0.52
1^st^	126	(41)	76	(42)	50	(41)	
2^nd^	91	(30)	50	(28)	41	(33)	
3^rd^	87	(29)	55	(30)	32	(26)	
Height (cm), means ±SD	158.1	±7.5	155.6	±5.6	161.9	±8.4	<0.01
Weight (kg), means ±SD	46.8	±7.4	45.8	±6.7	48.3	±8.1	<0.01
(Current weight – Ideal weight)/Ideal weight (%), means ±SD	−5.9	±10.2	−6.1	±10.7	−5.7	±9.5	0.73
Current body size^*^,means ±SD	4	±1.0	4.3	±1.1	3.7	±0.9	<0. 01
Ideal body size^*^,means ±SD	3.7	±0.8	3.4	±0.7	4.1	±0.7	<0.01
Current – Ideal^*^, n (%)							<0.01
1<	49	(16)	45	(25)	4	(3)	
1 or 0	255	(84)	136	(75)	119	(97)	
Extracurricular activities, n (%)							<0.01
Sports	185	(61)	95	(53)	90	(73)	
Non-physical	63	(21)	55	(31)	8	(7)	
None	54	(18)	29	(16)	25	(20)	
Numbers of skipping breakfast, n (%)							0.88
At least once in a week	38	(13)	23	(13)	15	(12)	
None	265	(87)	157	(87)	108	(88)	
Calorie-restricted diet, n (%)							<0.01
Yes	32	(11)	31	(17)	1	(1)	
Never	272	(89)	111	(83)	114	(99)	
*Self-esteem, means ± SD*							
Total score (min 4-max 20)	13.3	±3	13.2	±3.1	13.5	±2.8	0.28
*Physical symptoms, means ± SD*							
Total score (min 4-max 12)	7.5	±2.1	7.6	±2.1	7.3	±2.1	0.32
*Psychological symptoms, means ± SD*							
Total score (min 4-max 8)	5	±1.6	5.1	±1.6	4.9	±1.6	0.48

* Participants chose the drawing numbers that best matched their current and their ideal body sizes from 1 (smallest) to 8 (largest).

Table 2 shows participant characteristics associated with body image self-discrepancy defined by current – ideal body image scores greater than “1.” Those who had body image self-discrepancy were more likely to participate in non-physical extracurricular activities rather than sports (*p*=0.03), skip breakfast (*p*=0.02), and have a calorie-restricted diet (*p*<0.01). The following three aspects of self-esteem were lower among those who had body image self-discrepancy than those who did not: “On the whole, I am satisfied with myself” (*p*=0.01); “I feel that I have a number of good qualities” (*p*=0.02); and “I feel that I’m a person of worth, at least on an equal plane with others” (*p*=0.04). Total self-esteem scores were lower (*p*=0.01), and physical and psychological symptoms, including stomachaches (*p*=0.02), fatigue (*p*<0.01), sleepiness (*p*<0.01), and depressive mood (*p*<0.01), were higher among those who had body image self-discrepancy.

**Table 2:** Factors associated with body image self-discrepancy defined by current – ideal body image >1: univariate logistic regression analyses.

	Body image self-discrepancy		*p*	Logistic regression model		
	(+)	(−)		Crude OR	95%CI	
	n(%)	n(%)			Upper	Lower
Age, mean ± SD	14.0±0.8	13.8±0.8		1.29	0.9	1.87
Gender			<0.01			
Female	45 (92)	136 (53)		9.84	3.44	28.17
Male	4 (8)	119 (47)		1	-	-
*Extracurricular activities*						
Non-physical	16 (33)	47 (19)		2.52	1.23	5.19
None	10 (21)	44 (17)		1.68	0.74	3.82
Sports	22 (46)	163 (64)	0.03	1	–	–
Numbers of skipping breakfast			0.02			
At least once in a week	11 (22)	27 (11)		2.43	1.12	5.31
None	38 (78)	227 (89)		1	–	–
Calorie-restricted diet			<0.01			
Yes	17 (35)	15 (6)		8.67	3.94	19.07
No	32 (65)	240 (94)		1	–	–
*Self-esteem, mean ± SD*						
Total score	12.2±3.5	13.5±2.9	0.01	0.86	0.78	0.96
*Physical symptoms, mean ± SD*						
Total score	8.3±1.9	7.3±2.1	<0.01	1.26	1.08	1.48
*Psychological symptoms, mean ± SD*						
Total score	5.7±0.2	4.9±0.1	<0.01	1.47	1.18	1.83

Participants chose the drawing numbers that best matched their current and their ideal body sizes from 1 (smallest) to 8 (largest), and body image self-discrepancy was defined by current – ideal body image >1. OR, odds ratio; 95%CI, 95% confidence interval. Physical symptoms include headache, stomachache, and sleepiness. Psychological symptoms include fatigue and depressive mood. Self-esteem, physical symptoms, and psychological symptoms are tested by total scores.

Logistic regression models showed that significant factors associated with the increased risk for body image self-discrepancy were female vs. male gender (OR, 9.84, 95% CI: 3.44–28.17; *p*<0.01), participating in non-physical extracurricular activities vs. sports (OR, 2.52, 95% CI: 1.23–5.19; *p*=0.04), skipping breakfast vs. always eating breakfast (OR, 2.43, 95% CI: 1.12–5.31; *p*=0.03), calorie-restricted diet experience vs. no experience (OR, 8.67, 95% CI: 3.94–19.07; *p*<0.01), and a one-unit increase in total score of physical (OR, 1.26, 95% CI: 1.08–1.48; *p*<0.01) and psychological (OR, 1.47, 95% CI: 1.18–1.83; *p*<0.01) symptoms. In contrast, self-esteem was associated with a decreased risk of body image self-discrepancy (OR, 0.86 for a one-unit increase in total score, 95% CI: 0.78–0.96; *p*<0.01).

Table 3 shows the results of multivariable stepwise logistic regression modeling with body image self-discrepancy defined as a difference between current and ideal body image greater than “1.” In all students, female gender (OR, 6.92, 95% CI: 2.33–20.51; *p*<0.01), a calorie-restricted diet (OR, 5.18, 95% CI: 2.22–12.05; *p*<0.01), and psychological symptoms (OR, 1.47, 95% CI: 1.15–1.87; *p*<0.01) were significantly associated with an increased risk of body image self-discrepancy. Specifically, for girls, a calorie-restricted diet (OR, 5.43, 95% CI: 2.29–12.86; *p*<0.01) and psychological symptoms (OR, 1.46, 95% CI: 1.13–1.90; *p*<0.01) were significant factors associated with an increased risk of body image self-discrepancy. In boys, skipping breakfast (OR 7.85, 95% CI: 1.02–60.54; *p*<0.05) was the only factor associated with an increased risk of such self-discrepancy.

**Table 3:** Factors associated with body image self-discrepancy (current−ideal body image >1) in all students and girls: Multivariate stepwise logistic models.

	All students	Girls
	OR (95%CI)	OR (95%CI)
*Gender*		
Female vs. male	6.92 (2.33–20.51)	–
*Calorie-restricted diet*		
Yes vs. No	5.18 (2.22–12.05)	5.43 (2.29–12.86)
*Psychological symptoms, mean ± SD*		
Total score	1.47 (1.15–1.87)	1.46 (1.13–1.90)

OR, odds ratio; 95%CI, 95% confidence interval. ^*^Stepwise modeling process first involves starting with no variables in the model and ending with the full model (all the predictor variables selected). We considered age, gender, extracurricular activities, skipping breakfast, a calorie-restricted diet, self-esteem, and physical and psychological symptoms as predictor variables. Self-esteem and physical and psychological symptoms were entered as total score.

Although data were not shown, sensitivity analyses were conducted with a difference between current and ideal body image greater than “2.” Because there were only 6 students (girls, 100%), univariate logistic regression analyses suggest consistent results with analyses with a difference greater than “1”: a calorie-restricted diet (OR, 9.14, 95% CI: 1.76–47.38; *p*<0.01) and psychological symptoms (OR, 2.20, 95% CI: 1.11–4.38; *p*<0.05).

## Discussion

Our findings demonstrate that an increased risk of body image self-discrepancy was associated with female gender, a calorie-restricted diet in the past month, and psychological symptoms. Although none of our participants had ever been clinically diagnosed with an eating disorder, it should be noted that some of the findings observed in our study are typical among individuals with clinical eating disorders. Thus, the discrepancy between self-perceived body image (i.e., how one believes his or her body looks) and ideal body image (i.e., how one wants his or her body to look) observed among healthy adolescents in our study may be closely linked with cognitive distortion regarding body size and shape, which is a core symptom of clinical eating disorders.

The prevalence of anorexia in Japan is currently increasing. Such high prevalence rates, not only in Japan but also in other parts of Asia, may be attributable to the influence of Westernization. Accordingly, beauty standards have become separate from personal values. Additionally, the film and fashion industries encourage a thin-ideal culture, which, in turn, fosters increases in body dissatisfaction, restrictive dieting, and eating disorders (Pike and Dunne, 2015). The promotion of beauty ideals may determine how young people perceive and value themselves, potentially resulting in body dissatisfaction and low self-esteem (Aparicio-Martinez et al., 2019). Evidence for the purported role of “Westernization” in engendering eating disorders in Asian populations includes research from Singapore (Chua et al., 2021), China, the United Kingdom, Spain (Agüera et al., 2017), and Taiwan (Chang et al., 2013), all indicating a correlation between the degree of thin-ideal internalization and increased body image self-discrepancy.

The proportion of those with body image self-discrepancy was remarkably different between boys and girls. In previous studies, body image self-discrepancy has been shown to be more prevalent in women than men, which is consistent with our finding that few boys had such self-discrepancy. Research on body mass and body image has largely focused on dieting, eating, eating disorders, and depressive symptoms, especially among women and girls (Cash, 2004). An interesting study using a sample of 448 boys and 508 girls in Melbourne, Australia, reported that for girls, body dissatisfaction reflected a predominant desire to be thinner; however, for boys, those who were overweight wanted to lose weight, whereas those who were underweight wanted to gain weight (Kostanski et al., 2004). Another study in Canada (Solomon-Krakus et al., 2017), conducted with 556 young adolescents (45% girls), found that both girls and boys who perceived themselves as having body shapes significantly larger than their ideal size experienced the highest levels of depressive symptoms relative to their peers. The authors suggested that there may be a linear association between one’s actual body size and depressive mood and posited that, for girls, this finding supported the common notion of a drive for thinness. However, for boys, the results were more complicated; although boys at a healthy weight were expected to want to gain weight and be more muscular, overweight boys were dissatisfied with their weight and wanted to be thinner, similar to their female counterparts. One possible explanation for this may be the participants’ ages (9–13years), as boys tend not to focus on muscularity until they reach puberty (McCabe and Ricciardelli, 2004). In a review of a large study with a representative sample of Dutch children aged 11 to 16years (ter Bogt et al., 2006), boys tended to compare themselves to the ideal of a lean, muscular body type, prompting anxiety about being either too thin or too overweight, even when their weight was within a healthy body mass index (BMI) range. Further, girls were shown to possibly have ideals of impeccable thinness, evoking fears of irreversible weight gain, even when their BMI was within a healthy range. During late adolescence, bodyweight dissatisfaction applies to approximately half of boys and the majority of girls, indicating that adolescence is indeed a period of intensified anxiety about the shape and size one’s body will take in adulthood (ter Bogt et al., 2006). Alternatively, dieting and a desire to lose weight may be particularly salient in households where at least one parent is obese (Solomon-Krakus et al., 2017), which may be more likely in Western countries, where obesity is much more prevalent than in Japan. These findings were consistent with studies conducted in other countries, indicating that a similar set of gender-specific cultural pressures regarding ideal body shape operates throughout the industrialized world (Adams et al., 1993; de Castro and Goldstein, 1995).

A significant amount of research on the relationship between eating disorders and psychopathology has confirmed the complexity of these problems and difficulties in diagnosis and treatment (Marucci et al., 2018). For example, a randomized study (Junne et al., 2016) conducted with 242 women with anorexia who were between 18 and 56years of age found that body image perceptions in early treatment stages predicted depression and anxiety in follow-up measurements; further, the predictive association persisted when BMI increased during treatment. The authors concluded that body image disturbances in patients with anorexia should, therefore, be explicitly targeted during specialized psychotherapy with affected patients.

A study suggested that young adolescents with body image self-discrepancy may benefit from cognitive-based training (Zipfel et al., 2014), and a previous intervention study (Armitage, 2012) demonstrated an increased level of self-affirmation reduced body dissatisfaction. The literature further suggests that increasing self-esteem, confidence, and self-worth may improve self-perception of body image and, consequently, may improve eating habits (Seekis et al., 2017; Moffitt et al., 2018). Although we did not observe an association between self-esteem and body image self-discrepancy, a future study with a larger sample is warranted to identify protective factors for body image self-discrepancy.

This study has some limitations that should be addressed. First, in addition to having a relatively small sample size, we collected data from only one of the 17 junior high schools located in Saitama, Kumagaya City; thus, the sample reflects only 10% of the city’s junior high school students. However, despite the small sample size, the prevalence of cognitive distortion related to body image found in our study was similar to the prevalence found in a larger study previously conducted in Japan (Shirasawa et al., 2016). The previous study investigated body image disturbance among 1,821 junior high school students from 2005 to 2009 (age: 12–13years) and reported that 18.4% of girls overestimated their weight. Although that study used self-perceived weight, which differs from our assessment method, the proportion of girls who overestimated their measured weight was very close to the proportion in our sample (i.e., 18.4% of 13-year-old girls).

Second, the average weight of girls in our sample (45.8kg for girls aged 13–15years) was slightly lower than that of the general population of the same age group in Japan. In fact, the published 2018 School Health Statistics in Japan reported that 2.78% of 14-year-old girls were underweight at that time; the percentage of underweight girls aged 13–15 in the present study was slightly higher, at 8.53% (School Health Statistics, 2018). Previously, Takimoto et al. (2004) reported that women in metropolitan areas were more likely to be underweight compared to women who resided in small/rural towns. Notably, Kumagaya City is one of the largest cities in Saitama prefecture, which is part of the greater metropolitan Tokyo area.

Third, we measured fatigue and depressive mood using only one self-report question rated using a Likert scale. A similar study in Canada (Solomon-Krakus et al., 2017) used the 12-item Centre for Epidemiological Studies Depression Scale; although we did not use such a validated scale, fatigue and depressive mood are very frequent and specific symptoms of major depression (Nakao and Yano, 2006). In fact, they are listed among the components of the diagnostic criteria for major depressive disorder in the *Diagnostic and Statistical Manual of Mental Disorders* (Fifth Edition).

Fourth, we abbreviated an established scale to measure self-esteem. Additionally, we assessed unhealthy eating behaviors and physiological/psychological symptoms with single-item questions, which might have made it more likely that their prevalence would be overestimated.

Fifth, our survey did not contain detailed questions regarding distorted cognition related to body size or sociodemographic information. In addition, we did not discuss the pathology of eating disorders within the context of genetic approaches, as previous literature has suggested (Duncan et al., 2017). Lastly, as this is a cross-sectional study, we could not determine causal relationships between body image self-discrepancy and the associated factors. Given these recognized limitations, the results of the present study require careful interpretation.

## Conclusion

This study was an investigation of body image self-discrepancy among young, healthy adolescents. We found that an increased risk of body image self-discrepancy was associated with female gender, calorie-restricted diets, and the psychological symptoms of fatigue and depressive mood. Given that clinical eating disorders are prevalent globally, it is necessary to provide effective early interventions for young adolescents to decrease the prevalence of body image self-discrepancy. Additionally, future studies are warranted to investigate whether body image self-discrepancy is a precursor to distorted cognition related to body size, a core manifestation of eating disorders.

## Data Availability Statement

The original contributions presented in the study are included in the article/supplementary material, further inquiries can be directed to the corresponding author.

## Ethics Statement

The studies involving human participants were reviewed and approved by the ethics committee at Teikyo Heisei University. Written informed consent to participate in this study was provided by the participants’ legal guardian/next of kin.

## Author Contributions

HK and KO conceived the study. KN and YI drafted the manuscript, which was critically reviewed and revised by SM. The data were collected by YS, YT, AN, KE, HT, YK, HA, YM, NK, SH, and HK. All the authors provided approval for publication of the content.

## Funding

This study was supported by the Ministry of Education, Culture, Sports, Science and Technology, Japan (Grants for Scientific Research [B], no. 16H05262).

## Conflict of Interest

The authors declare that the research was conducted in the absence of any commercial or financial relationships that could be construed as a potential conflict of interest.

## Publisher’s Note

All claims expressed in this article are solely those of the authors and do not necessarily represent those of their affiliated organizations, or those of the publisher, the editors and the reviewers. Any product that may be evaluated in this article, or claim that may be made by its manufacturer, is not guaranteed or endorsed by the publisher.
